# Zahedan rhabdovirus, a novel virus detected in ticks from Iran

**DOI:** 10.1186/s12985-015-0410-5

**Published:** 2015-11-05

**Authors:** Meik Dilcher, Oumar Faye, Ousmane Faye, Franziska Weber, Andrea Koch, Chinikar Sadegh, Manfred Weidmann, Amadou Alpha Sall

**Affiliations:** Department of Virology, Univerity Medical Center Göttingen, Kreuzbergring 57, 37075 Göttingen, Germany; Institute Pasteur de Dakar, 36 Avenue Pasteur, BP 220 Dakar, Senegal; Pasteur Institute of Iran, 69 Pasteur Avenue, Tehran, Iran; Institute of Aquaculture, University of Stirling, Stirling, FK9 4LA UK

**Keywords:** Zahedan rhabodovirus, ZARV, Tick-transmitted, *Hyalomma anatolicum*, Iran

## Abstract

**Background:**

*Rhabdoviridae* infect a wide range of vertebrates, invertebrates and plants. Their transmission can occur via various arthropod vectors. In recent years, a number of novel rhabdoviruses have been identified from various animal species, but so far only few tick-transmitted rhabdoviruses have been described.

**Methods:**

We isolated a novel rhabdovirus, provisionally named Zahedan rhabdovirus (ZARV), from *Hyalomma anatolicum anatolicum* ticks collected in Iran. The full-length genome was determined using 454 next-generation sequencing and the phylogenetic relationship to other rhabdoviruses was analyzed. Inoculation experiments in mammalian Vero cells and mice were conducted and a specific PCR assay was developed.

**Results:**

The complete genome of ZARV has a size of 11,230 nucleotides (nt) with the typical genomic organization of *Rhabdoviridae*. Phylogenetic analysis confirms that ZARV is closely related to Moussa virus (MOUV) from West Africa and Long Island tick rhabdovirus (LITRV) from the U.S., all forming a new monophyletic clade, provisionally designated *Zamolirhabdovirus*, within the *Dimarhabdovirus* supergroup. The glycoprotein (G) contains 12 conserved cysteins which are specific for animal rhabdoviruses infecting fish and mammals. In addition, ZARV is able to infect mammalian Vero cells and is lethal for mice when inoculated intracerebrally or subcutaneously. The developed PCR assay can be used to specifically detect ZARV.

**Conclusion:**

The novel tick-transmitted rhabdovirus ZARV is closely related to MOUV and LITRV. All three viruses seem to form a new monophyletic clade. ZARV might be pathogenic for mammals, since it can infect Vero cells, is lethal for mice and its glycoprotein contains 12 conserved cysteins only found in animal rhabdoviruses. The mammalian host of ZARV remains to be identified.

## Background

Rhabdoviruses are non-segmented, negative-sense RNA viruses with 11 to 15 kilobasepair (Kb) genomes coding for at least five transcription units: the nucleoprotein (N), phosphoprotein (P), matrix protein (M), glycoprotein (G) and the RNA-dependent RNA-Polymerase (L) each flanked by conserved transcription initiation and termination/polyadenylation signals and a short intergenic region. In the genomes of some genera of the *Rhabdoviridae* additional genes are interposed between the five canonical protein genes. Some rhabdoviruses contain alternative or overlapping ORFs within the five main coding regions that may encode for additional accessory proteins, most without known function and homology to any other known proteins [1].

The *Rhabdoviridae* include species infecting a wide range of vertebrates, invertebrates and plants [2]. To date the complete genomes of more than 80 rhabdoviruses have been identified which can be phylogenetically grouped into eleven genera (*Lyssavirus, Vesiculovirus, Ephemovirus, Novirhabdovirus, Cytorhabdovirus, Nucleorhabdovirus, Perhabdovirus, Sigmavirus, Sprivivirus, Tibrovirus and Tupavirus*) and at least two additional proposed genera (*Hart park group and Almpivar group* [3] not yet officially recognized by the ICTV.

The advent of next generation sequencing has led to the determination of numerous novel rhabdovirus genomes from bats as Bokeloh bat lyssavirus from *Myotis nattereri* in France [4] or Fikirini rhabdovirus from *Hipposideros commersoni*, in Kenya [5], from pigs in China [6], from kudu (*Tragelaphus strepsiceros*) in Namibia [7], or humans in Korea [8].

Four novel rhabdoviruses (Tench rhabdovirus, Grass carp rhabdovirus, Perch rhabdovirus and Eel rhabdovirus) isolated from fish and members of the genera *Sprivivirus* and *Perhavirus* were described [9], as well as Bas-Congo virus, a putative member of the genus *Tibrovirus* associated with acute hemorrhagic fever in humans in Central Africa [10]. Additionally, other novel rhabdoviruses, which are distinct from, and group outside of any of the existing eleven genera and two putative genera such as Farmington virus, a novel bird rhabdovirus [11] were described.

Rhabdovirus transmission can occur via arthropod vectors, including mosquitoes, midges, sandflies, ticks, aphids and leafhoppers [2]. Recent work describing Beaumont and North Creek virus isolated from mosquitoes in Australia places them in the dipteran-mammal associated *Dimarhabdovirus* supergroup comprised of the genera *Vesiculovirus*, *Sprivivirus*, *Perhavirus*, *Tibrovirus*, *Ephemovirus*, *Sigmavirus*, *Tupavirus* and several unassigned viruses which apparently cycle between diptera and mammalia [2, 3, 12]. This supergroup also contains the recently described Arboretum and Puerto Almendras viruses isolated from mosquitoes collected in Peru [13], and Yug Bogdanovac virus isolated from a pool of *Phlebotomus perfiliewi* sandflies collected in Serbia [14].

Additionally, rhabdovirus genomes derived of isolates from arthropods distinct from any of the approved phylogenetic groups such as that for Niakha virus isolated from phlebotomine sandflies in Senegal [15], Kolente virus, isolated from ticks and bats in the Republic of Guinea [16], Moussa virus (MOUV), isolated from *Culex decens* mosquitoes in Côte d’Ivoire [17] and Long Island tick rhabdovirus (LITRV) isolated from *Amblyomma american*um ticks [18] in the USA were characterized.

Here we describe the genome of a new rhabdovirus, provisionally named Zahedan rhabdovirus (ZARV), isolated from a *Hyalomma anatolicum anatolicum* tick from Iran, which together with MOUV and LITRV appears to form a new monophyletic group of distinctly arthropod-borne rhabdoviruses.

## Results

### Identification and genome organization

Homogenates of 11 ticks collected in Iran were inoculated on 11 separate Vero cell cultures and a typical cytopathic effect indicated virus growth in one culture (Ar Teh 157764). The isolate was subjected to 454 FLX pyrosequencing. *De novo* assembly and blastx analysis of the 246.016 reads obtained from a MID-tagged Rapid Library detected the genome of a new rhabdovirus, provisionally designated Zahedan rhabdovirus (ZARV). Almost the complete genome (99 %) was determined and the 3′ leader and 5′ trailer sequences were recovered via Sanger sequencing of amplicons generated with primers complementary to the 3′ and 5′ adapters in combination with specific internal primers (see Methods). Only 4 % (10.406 reads) of the 246.016 total reads were specific for the new rhabdovirus, still yielding an average genome coverage of about 319-fold. The remainder of the reads contained sequences from the cell-culture cells used for virus isolation (95.86 %) and mycoplasma sequences (0.14 %).

**Fig. 1:**
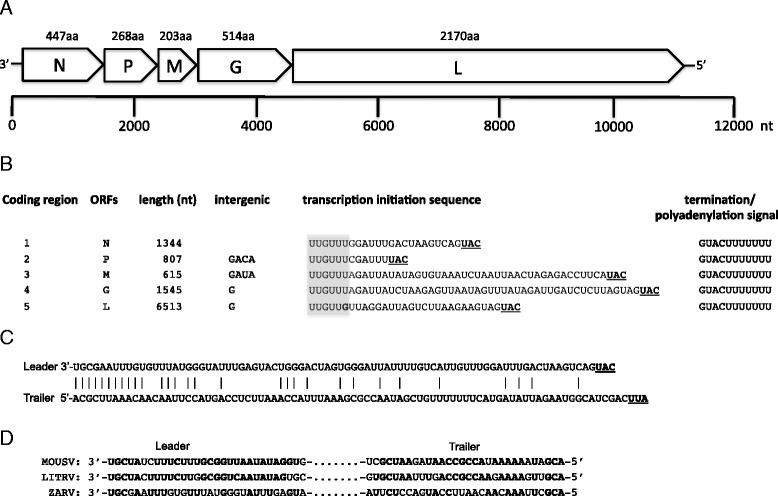
Scheme of the genome organization of ZARV. **a** Schematic representation of ZARV ORFs. **b** ZARV coding regions and corresponding transcription regulatory sequences. **c** Complementarity of the leader and trailer of ZARV. The complement of start and stop codons is underlined. **d** Alignment of the leader and trailer sequences of MOUV, LITRV and ZARV. Conserved nucleotides are indicated in bold

The ZARV genome consists of one segment of negative-stranded ssRNA with a size of 11,230 nt (GenBank accession: KJ830812) and the typical genomic organization of *Rhabdoviridae*, including a 3′ leader followed by 5 open reading frames (ORFs, Fig. 1a) each flanked by conserved transcription initiation (UUGUUU/G) and transcription termination/polyadenylation signals (GUAC[U]_7_) separated by 1–4 intergenic nucleotides, and a 5′ trailer sequence (Fig. 1b). There were no additional genes interposed between the major protein genes, as found in some genera of the *Rhabdoviridae* [19]. All additional ORFs were shorter than 180 nt producing putative polypeptides < 60 amino acids (aa) with no conserved domains found via the NCBI Conserved Domains Database search [20], and no homology to any other viral protein by blastp analysis.

A closer investigation of the ZARV genome indicated that three ORFs (1, 4 and 5) show homology to rhabdoviral N, G and L proteins with 29.1, 24 and 45.4 % sequence similarity to MOUV proteins in a ClustalW pairwise sequence alignment, respectively (Table 1). ORF2 showed no nt or aa homology in blastn and blastp search to viral sequences deposited in GenBank and ORF3 only showed 28 and 25 % aa identity to the ORF3 proteins of LITRV and MOUV via blastp search on NCBI, respectively. In the ClustalW pairwise sequence alignment, the ORF-2 encoded putative phosphoprotein shows only 15.1 and 13.6 % similarity to the phosphoproteins of MOUV and LITRV, respectively. The putative ORF-3 matrixprotein shows 18.4 and 23.0 % identity to the respective matrixproteins of MOUV and LITRV (Table 1).

**Table 1:** Comparison of ZARV, MOUV and LITRV proteins. Percent amino acid identity after pairwise ClustalW alignment using MegAlign 10.1.0 to ZARV or between MOUV and LITRV are indicated

ORF	ZARV (KJ830812)	MOUV (FJ985749)	LITRV (KJ396935)	MOUV/LITRV
N	447 aa	467 aa (29.1 %)	470aa (24.9 %)	37.5 %
P	268 aa	289 aa (15.1 %)	276 aa (13.6 %)	22.3 %
M	203 aa	241 aa (18.4 %)	205 aa (23.0 %)	32.7 %
G	514 aa	526 aa (24.0 %)	506 aa (21.6 %)	29.7 %
L	2170 aa	2141 aa (45.4 %)	2131 aa (44.9 %)	51.9 %

A common feature of rhabdovirus genomes is the complementarity of the 3′ leader and the 5′ trailer sequence [21]. The 3′ leader of ZARV consists of 79 nt and the 5′ trailer of 84 nt with 30 nt being complementary. Within the first 20 terminal nt, 16 nt show complementarity (Fig. 1c). In rhabdoviruses known to infect mammals, the first three nucleotides (UGC) and the tenth nucleotide (U) are usually conserved [17], as can be seen in MOUV and LITRV. ZARV does contain the conserved UGC sequence but instead of the conserved U it contains a G at position ten (Fig. 1d).

**Fig. 2:**
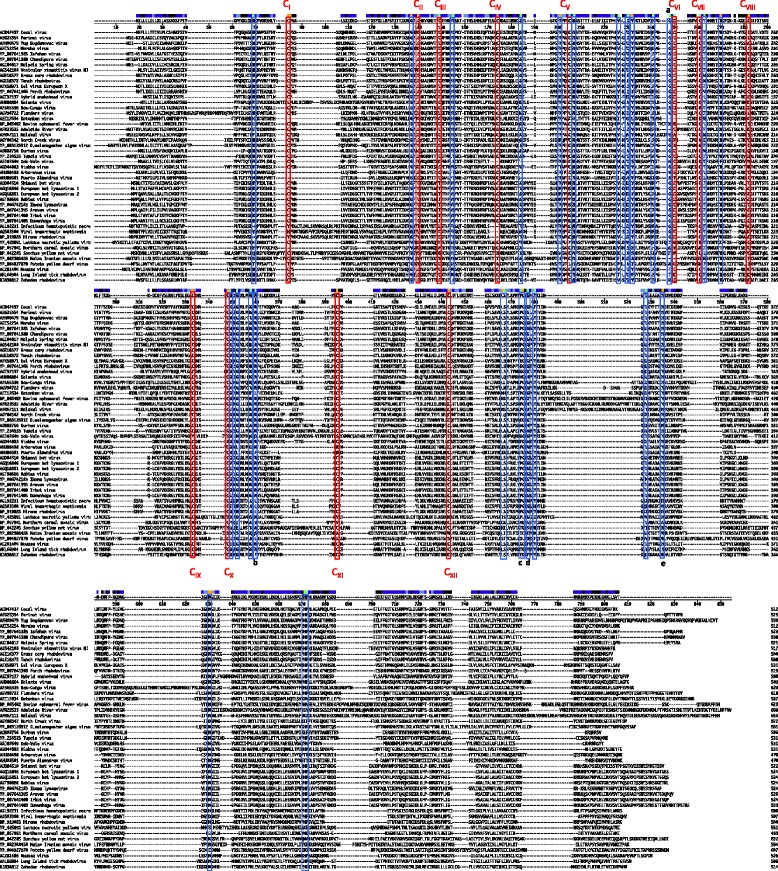
ClustalW alignment using MegAlign 10.1.0 (DNASTAR) of the deduced amino acid sequences of the G proteins of 47 selected rhabdoviruses including animal and plant viruses. Highly conserved cysteine residues have been numbered sequentially (C_I_ to C_XII_) and labeled in red bars [1]. Positions of universally conserved amino acids are shown in blue bars. For ZARV characteristic amino acid exchanges of universally conserved positions are indicated with letters a-e

The only surface protein for rhabdoviruses, glycoprotein (G), binds to cellular receptors. It therefore plays an important role in tropism and pathogenicity. ZARV contains 19 cysteines of which 12 comprise the conserved cysteines found in animal rhabdoviruses (C_I_ to C_XII_, [1]) infecting fish and mammals. In addition to these conserved cysteine residues, rhabdovirus glycoproteins contain several other conserved amino acid positions, which might be involved in [22] host tropism. ZARV displays the amino acid substitutions G171D, W249F, P321E, G324F and Y341W in these additional conserved positions compared to the consensus sequence of rhabodvirus glycoprotein sequences (Fig. 2). The W249F exchange is shared by Puerto Almendras virus, the P321E exchange is shared by Eel virus European X, Perch rhabdovirus, D. melanogaster sigma virus and Niakha virus, the G324F exchange is shared by MOUV and the Y341W exchange is shared by Maize Iranian mosaic virus and Potato yellow dwarf virus.

### Phylogeny

**Fig. 3:**
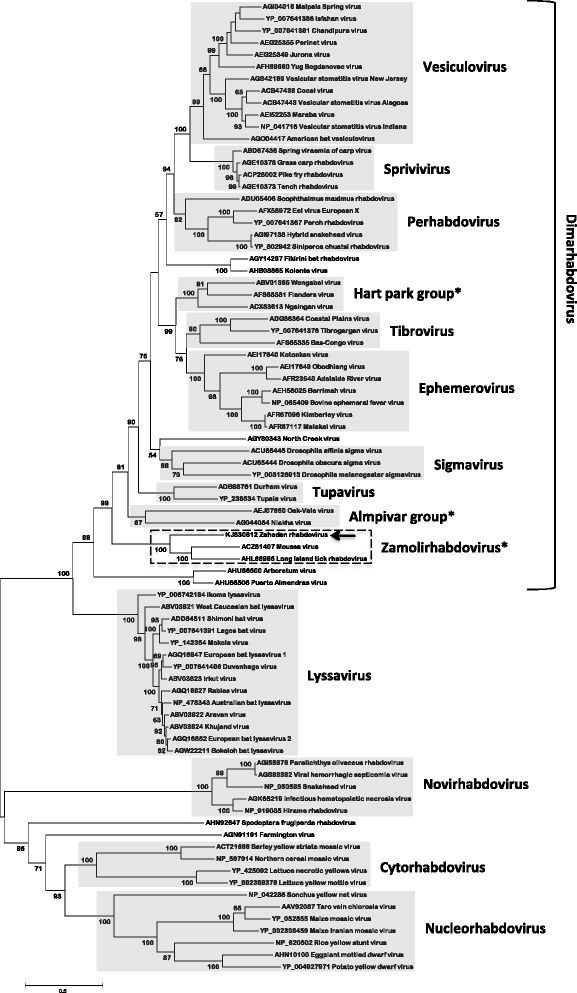
Maximum-likelihood (ML) phylogenetic tree based on 81 complete rhabdovirus L protein amino acid sequences aligned via MUSCLE and calculated using MEGA 5.2.2. Bootstrap values (>50 %) are given in percent. The scale bar indicates number of substitutions per site. * Genera proposed but not yet formally approved by the International Committee on Taxonomy of Viruses (ICTV). The arrowhead indicates Zahedan rhabdovirus (ZARV) and the dashed box indicates the proposed new rhabdovirus genotype Zamolirhabdovirus

Since the L protein is the most conserved rhabdovirus protein, especially in a conserved sequence motif designated block III [3], we performed a MUSCLE alignment of 81 different rhabdovirus L protein sequences. Fig. 3 shows a Maximum-likelihood (ML) phylogenetic tree based on this alignment. The robustness of each node was determined using 1000 bootstrap replicates. Here, ZARV forms a monophyletic group together with MOUV and LITRV.

### Growth characteristics

**Fig. 4:**
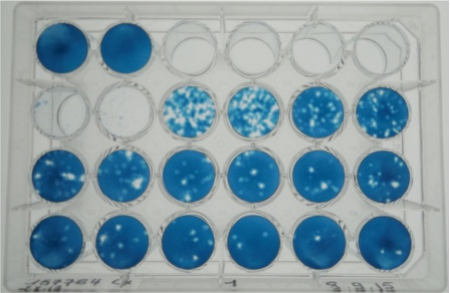
ZARV plaque assay: A1-2: negative control (cell culture medium). A3-D6: serial 10-fold dilutions in duplex wells down to 10^−11^ of ZARV culture supernatant on Vero cell culture monolayers. The ZARV inoculum as well as the Vero cells was previously confirmed by PCR to be negative for mycoplasma sequences (data not shown)

After inoculation of Vero cell cultures, ZARV (Ar Teh 157764) developed a CPE within 3 days, indicating virus growth. No CPE was observed in the negative control culture (Fig. 4). The ZARV inoculum was tested negative for mycoplasma sequences using PCR (data not shown).

In addition, newborn (1–2 day old) mice were inoculated with 20 μl Vero-grown ZARV containing approximately 2x10^7.6^ lethal dose 50% (LD50) per ml by intracerebral, intraperitoneal and subcutaneous route. The animals were observed daily for illness. Animals were tested for ZARV infection by PCR. The intracerebral inoculation route showed infection of the animals at 4 and 6 days postinoculation (8/10 = 80 %). No illness was observed after subcutaneaous and intraperitoneal inoclulation. The intracerebral inoculation route showed such severe symptoms that the mice had to be euthanized at 4 and 6 days postinoculation (8/10 mice = 80 %). No disease was observed after subcutaneous and intraperitoneal route inoculation.

### Development of a classical PCR assay

We developed a conventional RT-PCR assay detecting an amplicon within the ORF2/putative phosphoprotein gene with primers ZARV-UP and ZARV-DP, which can be used to specifically identify ZARV. This assay was used to retest the supernatants of all eleven virus isolation attempts (all samples were collected at the same time in the same area of Iran), confirming the ZARV positive supernatant but not detecting any other positive supernatants (data not shown). In addition, this assay was used to test ZARV-RNA = Ar Teh 157764 [Zahedan/Iran 2001] and in parallel 24 African rhabdovirus RNA’s from viruses isolated in the years 1959 until 2013: MOSSURIL [Mozambique 1959], RV401 (AND401) [Senegal 1964], ANB373 [Central African Republic 1970], SA262037 [Senegal 2013], SA194858 [Senegal 2008], SA217694 [Senegal 2011], SA206776 [Senegal 2010], ARD111213 [Senegal 1995], ARD129179 [Senegal 1997], ARD111757 [Senegal 1995], MOULIN [n.d.], CHAND13 [n.d.], AND42443 [Senegal 1985], LG6 [n.d.], YM55 [Cameroune 1965], ASH763 [Senegal 1965], ANB439 [Central African Republic 1970], MOKOLA [Cameroune 1973], ANB1094 [Central African Republic 1970], ANB4289 [Central African Republic 1973], ARD129178 [Senegal 1997], ARD1275 [n.d.], RV5314 [Senegal 1968], ANK6909 [Guinea 1985]. Only the ZARV-RNA produced a specific band at 807 bp (data not shown). Furthermore, all the brain tissues of the deceased mice tested positive with this RT-PCR.

## Discussion

Phylogenetic analysis of the genome sequence groups ZARV into a monophyletic clade with LITRV another tick-borne virus from the U.S.A. and MOUV a mosquito-borne virus from Côte d’Ivoire.

Although G proteins of rhabdoviruses from different genera only share very low amino acid sequence identity, alignment of the sequences reveals remarkable conservation of cysteine residues, glycosylation sites and the major antigenic domains [1]. Since the glycoproteins interact with specific cell-surface receptors of the host cells sequence analysis might give indications about the putative animal host and the host surface-receptor usage of the respective rhabdovirus.

Twelve of nineteen cysteines found in the ZARV G sequence are typically conserved in animal rhabdoviruses (C_I_ to C_XII_, [1]) indicating that the new tick-transmitted rhabdovirus does not seem to be a pure tick virus (Fig. 2). These 12 cysteines are also conserved in MOUV and LITRV. Some of these conserved cysteines are not present in Niakha virus (lacking C_VIII_ and C_X_) isolated from phlebotomine sandflies or Drosophila melanogaster sigmavirus (lacking C_VII_) both without any known vertebrate host, or in plant rhabdoviruses like Maize Iranian mosaic virus (lacking C_II_, C_III_, C_IV_, C_VI_, C_VII,_ C_IX,_ C_X_). In addition, the mosquito-borne rhabdoviruses Arboretum virus and Puerto Almendras virus are lacking C_I_, C_VI_ and C_XI_. It needs to be further analyzed if these substitutions play a role in host tropism or if one can predict a potential host based on the amino acid composition of these conserved positions.

Interestingly, ZARV shows the highest aa similarity in N, P, G and L proteins to the mosquito-borne MOUV, with 29.1, 15.1, 24.0 and 45.4 % identity, respectively (Table 1). Only the M protein shows a higher similarity (23.0 %) to the tick-transmitted LITRV. But in general, MOUV and LITRV are more closely related and ZARV seems to represent an ancestral relative of both viruses, which is supported by high bootstrap values. This also features in the termination/polyadenylation sequence GAAC[U]_7_ which is identical for MOUV and LITRV, but slightly different for ZARV which uses GUAC[U]_7_.

The virus was cultured in cell culture and in mouse brain where it grew to high titres of 2x10^7.6^ LD50 typical for rhabdoviruses. Here it led to severe symptoms indicating pathogenicity in 80 % of the mice, so that they had to be euthanized 6 or 8 days postinoculation. We developed a conventional RT-PCR assay, which can be used to specifically identify ZARV.

In summary it can be said that although the tick-borne rhabdovirus subclade into which ZARV groups is phylogenetically distinct from the major rhabdovirus groups, the overall pattern of conserved amino acids of the G proteins of ZARV, MOUV and LITRV support their association to the (*Dimarhabdovirus* supergroup. Since all three viruses form a monophyletic group we suggest to combine them in a new genus designated *Zamoli*rhabdovirus (*Za*hedan rhabdovirus, *Mo*ussa virus, *L*ong *I*sland tick rhabdovirus). It remains to be seen if mammalian hosts can be identified for this new rhabdovirus genus.

## Materials and methods

ZARV was isolated on the 21st of December 2001 at the Institute Pasteur de Dakar, Senegal from a *Hyalomma anatolicum anatolicum* tick collected on the 27th of October 2001 in Zahedan/Iran. In this attempt, 11 homogenized ticks (150 μl each) were inoculated separately onto confluent Vero cell culture monolayers (25 cm^2^) in Leibovitz 15 growth medium (L-15) supplemented with 5 % fetal bovine serum (both GibcoBRL, Grand Island, NY, USA), and 10 mM each of Penicillin and Streptomycin (Sigma, Gmbh, Germany).

At 1 hpi, cells were washed twice with PBS and incubated with fresh medium. At 7 dpi, supernatant of infected cells was harvested and used for sub-passaging. After appearance of a CPE in one of the cultures (3 dpi) passage 6 was harvested and 140 μl of supernatant was used for RNA extraction via the QIAamp viral RNA mini kit (Qiagen), omitting the addition of carrier RNA. After DNase digestion (Turbo-DNA-Free-Kit, Ambion) and Pellet-Paint precipitation (Pellet Paint NF Co-Precipitant, Novagen), the purified RNA was used for amplification via the TransPlex Whole Transcriptome Amplification Kit (WTA2) from Sigma-Aldrich [23]. After purification via the QIAquick PCR purification kit (Qiagen), an additional size exclusion step via Ampure XP-beads (Agencourt) was used to remove fragments shorter than 350 bp. 300 ng of the whole genome amplified dsDNA was used for Roche Rapid Library Preparation employing MID barcodes starting with the End Repair step [24]. The MID-tagged Rapid Library was sequenced in a pool of 11 MID-tagged libraries. For determining the 3′ leader region, a 3′-FLAC adapter was ligated to the 3′-OH group [25]. A PCR with a complementary primer to the FLAC adapter in combination with an internal primer and sanger sequencing of the PCR product yielded the 3′ leader. To determine the 5′ trailer, first an RNA-5′-polyphosphatase treatment (Epicenter Biotechnologies) was carried out to remove two phosphate groups of the 5′-triphosphate. Next a 5′-RACE adapter (FirstChoice RLM-RACE kit, Ambion) was ligated to the 5′-monophosphate and a PCR with an 5′-RACE-outer primer in combination with an internal primer and sanger sequencing of the amplicon closed the gap on the 5′ trailer. Assembly of the genome was done with the GS De Novo Assembler (Newbler) version 2.6 in combination with the SeqMan Pro Software version 10.1.0. Phylogenetic analysis was performed using complete L protein sequences downloaded from GenBank and aligned to the L protein sequence of ZARV via ClustalW using MegAlign 10.1.0 (DNASTAR) and MUSCLE using MEGA 5.2.2 [26]. A maximum likelihood tree was generated using the Jones-Taylor-Thornton model with MEGA 5.2.2. Statistical significance of the tree topologies was evaluated by 1000 bootstrap replicates.

### Mouse experiments

Mice used in this study were reared at the Institute Pasteur in Dakar animal laboratory accredited by WHO as Collaborating Centre for Arboviruses and/or Hemorrhagic Fever Reference and Research and were used for routine virus isolation in suckling mice for surveillance, diagnostics and research as authorized by the senegalese national ethical comittee.

A serial dilution (10-^1^ to 10-^11^) of the Ar Teh 157764 strain was prepared in maintenance medium (L-15, with 3 % serum). Twenty microliter of each dilution of the virus was inoculated intracerebrally, intraperitoneal and subcutaneous into 10 BALB/c mouse pups (1–2 days old) provided by the Pasteur Institute Dakar facilities. The animals were observed for 21 days and ill mice were tested for ZARV infection using the PCR developed. The infectivity titer which is the reciprocal of the highest dilution showing 50 % mortality in the inoculated mice and expressed as LD_50_ /ml was calculated by using a formula similar to that described by Karber.

### Development of PCR assay

**Fig. 5:**
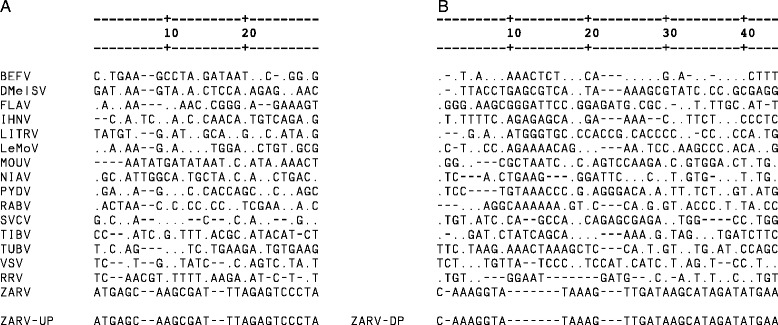
ZARV primer sequence alignment with other representative Rhabdovirus sequences, flanking the putative phosphoprotein/ORF2 gene. **a** alignment of upstream primer. **b** alignment of downstream primer. ZARV genome and primer sequences are represented as given. Nucleotides of the other Rhabdovirus sequences represented in the letter code differ from the ZARV sequence. Consereved nucleotides are represented as dots. BEFV: Bovine ephemeral fever virus (KM276084.1), DMelSV: Drosophila melanogaster sigma virus (NC_013135.1), FLAV: Flanders virus (KJ958896.1), IHNV: Infectious hematopoietic necrosis virus (NC_001652.1), LITRV: Long Island tick rhabdovirus (NC_025340.1), LeMoV: Lettuce yellow mottle virus (NC_011532.1), MOUV: Moussa virus (NC_025359.1), NIAV: Niakha virus (NC_025405.1), PYDV: Potato yellow dwarf virus (NC_016136.1), RABV: Rabies virus (AF499686.2), SVCV: Spring viraemia of carp virus (NC_002803.1), TIBV: Tibrogargan virus (GQ294472.1), TUBV: Tupaia virus (NC_007020.1), VSV: Vesicular stomatitis virus (NC_001560.1), PRV: Perch rhabdovirus (NC_020803.1). The alignment was calculated using MegAlign 10.1.0 (DNASTAR) employing the ClustalW algorithm

We developed a RT-PCR specific for ZARV, based on the phosphoprotein gene showing very low homology to phosphoprotein genes of other rhabdoviruses (Fig. 5). The RT-PCR with upper primer ZARV-UP: 5′-ATGAGCAAGCGATTTAGAGTCCCTA-3′ and lower primer ZARV-DP: 5′-TTAATTCATATCTATGCTTATCAACTTTA TACCTTTG-3′ generates an amplicon of 807 bp within the ORF2/putative phosphoprotein gene (data not shown).

In a 2 step RT-PCR reverse transcription was performed using M-MLV (Invitrogen, Carlsbad, USA) in a 20 μl mixture containing 4 μl first-strand buffer, 2 μl dithiothreitol, 1 μl RNAsin (40 U/μl) (Promega, Madison USA), 1 μl of M-MLV -RT enzyme incubated at 55 °C for 50 min, followed by 70 °C for 15 min. PCR was performed in a 50 μl volume using a commercial kit (Taq DNA polymerase, Promga, USA). Five microliters of cDNA was mixed with 5× Buffer, 100 nM each of forward and reverse primer, 100 μM dNTPs, 1.5 mM MgCl_2_, and 0,5 μl of enzyme Taq polymerase. The following thermal profile was used in GeneAmp 9700 (ABI, Singapore): 5 min at 95 °C followed by 40 amplification cycles of 1 min at 95 °C, 1 min 53 °C and 1 min 72 °C, and a final elongation step of 10 min 72 °C. Amplicons were analyzed in a 1 % agrose gel.

### Plaque assay

Supernatants from viral stocks were titrated on mycoplasma-free Vero cells (African Green Monkey Kidney) supplemented with 10 % fetal bovine serum (FBS) as well as penicillin-streptomycin (1 %) and fungizone (0,05 %). Viral supernatants were serially 10-fold diluted in L-15 medium with 10 % FBS. Two hundred microliters were inoculated on Vero cell monolayers in the wells of a 24-well plate. After 4 h of virus adsorption at 37 °C, cells were overlaid with 3,2 % of carboxymethylcellulose–L-15 medium containing 10 % FBS. After incubation at 37 °C for 4 days, cells were stained with 1 % black amido, dried at room temperature and the plaques were assessed (Fig. 4).
